# Antifungal activity of the culture filtrate of *Chaetomium subaffine* LB-1 against *Bipolaris maydis* and its underlying interaction mechanism

**DOI:** 10.3389/fmicb.2026.1848272

**Published:** 2026-05-20

**Authors:** Caiyun Liu, Zhenzhen Zhao, Zhilong Chang

**Affiliations:** 1 College of Advanced Agricultural and Life Sciences, Weifang University, Weifang, China; 2 Center of Teaching Quality Monitoring and Assessment, Weifang University, Weifang, China

**Keywords:** antifungal activity, *Bipolaris maydis*, *Chaetomium subaffine* LB-1, detoxification activity, mycolytic effect, nutrient uptake and metabolism

## Abstract

**Objective:**

*Chaetomium subaffine* LB-1 is a biocontrol strain identified in our laboratory. This study aimed to determine the antifungal activity of the culture filtrate of *C. subaffine* LB-1 against *Bipolaris maydis* and elucidate the pathogen response mechanism.

**Methods:**

Antifungal activity of the culture filtrate of *C. subaffine* LB-1 was assessed using a poison plate assay and a potted maize plant assay. The interaction mechanism between the culture filtrate and pathogen was further investigated through morphological observation, cell membrane permeability detection, and transcriptome analysis.

**Results:**

*In vitro* assays revealed that the culture filtrate of *C. subaffine* LB-1 significantly inhibited the growth of *B. maydis*, induced hyphal swelling and impaired cell membrane permeability of the pathogen. The potted maize plant assay confirmed the antifungal activity of the culture filtrate, achieving a control efficacy of 71.34% against southern corn leaf blight. RNA-seq analysis identified 4,124 differentially expressed genes (DEGs) in *B. maydis* treated with the culture filtrate (Bip_LB) compared with the untreated control (Bip_CK), including 1,924 upregulated and 2,200 downregulated genes. Combined gene function and metabolic pathway analyses of the DEGs indicated that genes associated with transmembrane transporter activity, carbohydrate degradation, amino acid degradation, DNA replication, and RNA synthesis and processing were significantly downregulated. In contrast, genes involved in phospholipid degradation, the pentose phosphate pathway, detoxification metabolism, and drug efflux were significantly upregulated.

**Conclusion:**

The culture filtrate of *C. subaffine* LB-1 exhibited strong antifungal activity and pronounced mycolytic effect against *B. maydis*. It impaired cell membrane permeability and cellular nutrient uptake, resulting in reduced carbohydrate and amino acid degradation and inhibited cell proliferation in *B. maydis*. Correspondingly, nutrient and energy compensation through lipid degradation and the pentose phosphate pathway, along with detoxification-related activities, were activated in *B. maydis* upon exposure to the culture filtrate of *C. subaffine* LB-1. These findings provide insights into the antifungal activity of the culture filtrate of *Chaetomium* species and support the application potential of the culture filtrate of *C. subaffine* LB-1 in management of southern corn leaf blight.

## 1 Introduction

*Bipolaris maydis*, the causal agent of southern corn leaf blight, poses a serious challenge to global maize production and can cause 9.7–11.7% yield losses (Singh and Srivastava, 2012; Aregbesola et al., 2020). Maize is the most widely cultivated grain crop in China, where southern corn leaf blight caused by *B. maydis* can lead to maize yield losses exceeding 30% in epidemic years in certain regions (Sun et al., 2020). Currently, the management of southern corn leaf bilight relies primarily on resistant cultivars and chemical fungicides. However, breeding resistant varieties is time-consuming and labor-intensive, and the extensive use of chemical fungicides may promote pathogen resistance and cause environmental pollution. Therefore, the development of eco-friendly biocontrol strategies is a promising approach for the management of southern corn leaf blight (Singh and Srivastava, 2012; Li et al., 2021).

*Chaetomium* spp. are ascomycetous fungi in the family Chaetomiaceae that have attracted considerable attention because of their biocontrol potential against plant diseases (Dwibedi et al., 2023; Lavanya et al., 2025). Antifungal activity of *Chaetomium* has been reported in several species, including *C. spirale* (Gao et al., 2005), *C. lucknowense* (Phong et al., 2016), and *C. globosum* (Zhang et al., 2021; Goswami et al., 2024; Lopez et al., 2026), etc. *Chaetomium* spp. can suppress plant pathogens through multiple mechanisms, among which antibiosis is the most widely discussed. Accordingly, the culture filtrates of several biocontrol *Chaetomium* strains, such as *C. globosum* CDW7 (Zhao et al., 2017), *C. globosum* DX-THS3 (Liao et al., 2024), and *C. globosum* M12XP1–2-3 (Feng et al., 2025), have been shown to exhibit strong antifungal activity. However, the antifungal mode of action of *Chaetomium* culture filtrates and the underlying mechanisms remain poorly understood. In addition, previous studies on the antifungal activity of *Chaetomium* culture filtrates have mainly focused on *C. globosum* and *Chaetomium* itself, whereas the stress response of target pathogens are often overlooked.

LB-1 is a biocontrol *Chaetomium* strain isolated from the leaves of *Sabina chinensis* cv. Kaizuka. It was taxonomically identified as *Chaetomium subaffine* based on morphological observation and internal transcribed spacer (ITS) sequence analysis by our research group (Liu et al., 2015). Previous studies have shown that the culture filtrate of *C. subaffine* LB-1 exhibits significant inhibitory effects against phytopathogenic fungi and induces mycolysis (Liu et al., 2021). Given the serious threat posed by southern corn leaf blight to maize production, *B. maydis* was selected as the target pathogen in this study to investigate the antifungal activity of the culture filtrate of *C. subaffine* LB-1, explore the underlying mode of action, and elucidate the stress response of the pathogen. This study provides valuable insights into the antifungal activity of *Chaetomium* spp. and highlights the application potential of the culture filtrate of *C. subaffine* LB-1 in the management of southern corn leaf blight.

## 2 Materials and methods

### 2.1 Fungal strains and culture medium

The biocontrol strain *Chaetomium subaffine* LB-1 and phytopathogenic fungus *Bipolaris maydis* race O were maintained in our laboratory. Both strains were cultured on potato dextrose agar (PDA) plates for 3 days prior to use in this study. Strain LB-1 was deposited in the China General Microbiological Culture Collection Center (No.11400).

### 2.2 Preparation of the culture filtrate of *C. Subaffine* LB-1

In preliminary experiments, the culture media and conditions for producing the culture filtrate of *C. subaffine* LB-1 with high antifungal activity have been screened and optimized using response surface methodology (RSM). Based on the optimized conditions, the culture filtrate of *C. subaffine* LB-1 was prepared as follows. 500-mL Erlenmeyer flasks containing 300 mL potato dextrose broth (PDB, containing 20% potato juice and 2% dextrose) were inocultaed with mycelial discs (6 mm in diameter) of strain LB-1 (10 discs per flask). The flasks were shaking culture at 25 °C and 130 rev·min^−1^ for 10 days. The culture broth was filtered through sterile gauze, and the filtrate was centrifuged at 4 °C and 8,000 *× g* for 15 min to remove any remaining spores and fungal mycelia. The supernatant was then passed through a 0.45 μm Millipore filter and used as the culture filtrate of *C. subaffine* LB-1(Xu et al., 2019; Bibi et al., 2023).

### 2.3 Determination of the inhibitory effect of the culture filtrate of *C. subaffine* LB-1 on *B. maydis*

#### 2.3.1 Poison plate assay

The *in vitro* inhibitory effect of the culture filtrate of *C. subaffine* LB-1 on *B. maydis* was determined using a poison plate assay. The culture filtrate of *C. subaffine* LB-1 was mixed with autoclaved PDA medium at a ratio of 1:2 (v/v) to prepare treatment plates. PDA medium without the culture filtrate was used as the control. Mycelial discs of *B. maydis* (6 mm in diameter) were excised from 3-day-old cultures and placed individually at the center of each plate. The plates were incubated at 25 °C for 6 days. Colony diameter and mycelial weight of *B. maydis* grown on treatment and control plates were measured to evaluate the antifungal effect of the culture filtrate. The inhibitory rate of the culture filtrate on the colony growth of *B*. *maydis* was calculated as follows: Inhibitory rate (%) = (1 − Dt/Dc) × 100, where Dt and Dc represent the mean colony diameters of *B*. *maydis* on the treatment and control plates, respectively. Three culture plates were included for each treatment and control.

#### 2.3.2 Potted maize plants assay

Potting soil was prepared by mixing field soil and organic nutrient-rich soil at a mass ratio of 3:1. Maize seeds (Shihai 928) were sown in pots and maintained in a greenhouse isolated from other plant pathogens under natural light at 17–30 °C and 60–70% relative humidity. When the plants reached the 6–8 leaf stage, one uniformly growing plant was retained in each pot and subjected to the following treatments: (1) foliar spraying with 10 mL of three-fold diluted culture filtrate of *C. subaffine* LB-1 at 24 h before inoculation; (2) foliar spraying with 10 mL of three-fold diluted culture filtrate of *C. subaffine* LB-1 at 24 h after inoculation; (3) foliar spraying with 10 mL of three-fold diluted culture filtrate of *C. subaffine* LB-1 at both 24 h before and 24 h after inoculation; (4) foliar spraying with 10 mL of PDB at 24 h after inoculation. Maize plants foliar sprayed with 10 mL of distilled water at 24 h after inoculation were used as the control. Inoculation was performed by spraying each plant with 5 mL of a *B. maydis* conidial suspension (1 × 10^5^ spore·mL^−1^). The conidial suspension was prepared by washing 8-day-old *B. maydis* cultures grown on PDA plates with phosphate-buffered saline and adjusting the concentration to 1 × 10^5^ spore·mL^−1^ using a hemocytometer. Ten pots were used for each treatment and control group individually. The disease index and control efficacy were calculated according to the Rules for Evaluation of Maize for Resistance to Pests, Part 2: Rule for Evaluation of Maize for Resistance to Southern Corn Leaf Blight (Agricultural Industry Standard of China, NY/T 1248.2–2006) as follows:

$$\begin{array}{l} \text{Disease index}\\ =\frac{\sum (\text{Number of diseased leaves}\times \text{Corresponding severity grade})}{\text{Total number of leaves}\times \text{Highest severity grade}}\times 100 \end{array}$$

$$\text{Control efficacy}\;(\%)=(\begin{array}{l} 1–\text{disease index of treatment}\\ /\text{disease index of control} \end{array})\times 100$$

### 2.4 Analysis of the mycolytic effect of the culture filtrate of *C. subaffine* LB-1 on *B. maydis*

#### 2.4.1 Morphological observation

The culture filtrate of *C. subaffine* LB-1 was diluted three-fold with PDB. Ten mycelial discs of *B. maydis* (6 mm in diameter) were inoculated into Erlenmeyer flask containing 100 mL of diluted culture filtrate and incubated on a rotary shaker at 25 °C and 130 rev·min^−1^. The cultured mycelia of *B. maydis* were observed microscopically at 24, 48, and 72 h post-inoculation (hpi). The mycelia of *B. maydis* cultured in PDB under identical conditions were used as the control. Three Erlenmeyer flasks were prepared for each treatment and control.

#### 2.4.2 Cell membrane permeability evaluation

The effect of the culture filtrate of *C. subaffine* LB-1 on the cell membrane permeability of *B. maydis* was evaluated based on the conductivity and leakage values of the culture supernatant, with reference to the method of Xu et al. (2019). Mycelial discs (6 mm in diameter) of *B. maydis* were inoculated into Erlenmeyer flasks containing 100 mL of three-fold diluted culture filtrate of *C. subaffine* LB-1 (10 discs per flask) and shake-cultured at 25 °C and 130 rev·min^−1^. Culture suspensions were collected individually at 0, 24, 48, and 72 hpi, centrifuged at 8,000 × *g* and 4 °C for 15 min. The resulting supernatants were used for conductivity and leakage assays. The conductivity was measured using a conductivity meter and calculated as [(J_1_ − J_0_)/(J_2_ − J_0_)] × 100, where J_1_ represents the conductivity of the culture supernatant, J_0_ represents the conductivity of the supernatant collected at 0 hpi, and J_2_ represents the conductivity of the boiled culture supernatant. The leakage was evaluated based on the OD_260_ values measured using a UV spectrophotometer. *B. maydis* cultured in PDB under identical conditions was used as the control. Three Erlenmeyer flasks were prepared for each treatment and control.

### 2.5 Transcriptome analysis of the interaction between the culture filtrate of *C. subaffine* LB-1 and *B. maydis*

#### 2.5.1 Sampling and RNA extraction

The culture filtrate of *C. subaffine* LB-1 was diluted three-fold with PDB. *B. maydis* shake-cultured in the diluted culture filtrate of *C. subaffine* LB-1 at 25 °C and 130 rev·min^−1^ for 48 h was used as the treatment sample (Bip_LB). *B. maydis* shake-cultured in PDB under the same conditions for 48 h was utilized as the control sample (Bip_CK). Three biological replicates were prepared for each treatment and control. The cultured mycelia of *B. maydis* were harvested and immediately frozen in liquid nitrogen for RNA extraction. Total RNA was extracted using the RNAprep Pure Plant Kit (TIANGEN, China), and RNA quality and concentration were assessed using the RNA Nano 6,000 Assay Kit on a Bioanalyzer 5,400 system (Agilent, United States).

#### 2.5.2 cDNA library construction, sequencing, and data analysis

cDNA library construction and sequencing were performed by Novogene Co., Ltd. (Beijing, China) using the PE150 mode on an Illumina NovoaSeq 6000 platform. The raw data (NCBI accession: PRJNA953188) were processed using FASTP (v0.19.7) to remove reads containing adapters, poly-N sequences, and low-quality bases (reads containing at least 50% of bases with Qphred scores ≤20). The clean reads were aligned to the reference genome of *B. maydis* (Ohm et al., 2012) using HISAT2 (v2.0.5) and assembled with StringTie (v1.3.3). Fragments per kilobase of transcript per million mapped fragments (FPKM) were calculated to estimate gene expression levels. Genes with FPKM > 1 in at least one sample were retained for subsequent analyses (Bray et al., 2016).

#### 2.5.3 Differentially expressed genes, GO function, and KEGG pathway analysis

DEGs between Bip_LB and Bip_CK were identified using DESeq2 (v1.20.0), with thresholds of |log_2_FoldChange| ≥ 1 and adjusted *p*-value (padj) ≤ 0.05 (Love et al., 2014). Functional annotation was performed by alignment against the reference genome, Swiss-Prot, and Pfam databases. Gene Ontology (GO) and Kyoto Encyclopedia of Genes and Genomes (KEGG) pathway enrichment analyses of the DEGs were conducted using the clusterProfiler R package (v3.8.1), with padj ≤ 0.05 as the significance threshold (Yu et al., 2012).

#### 2.5.4 Validation of DEGs by quantitative real-time PCR (qRT-PCR)

Gene expression profiles obtained from transcriptome analysis were validated using qRT-PCR. Mycelial samples of *B. maydis* were prepared for total RNA extraction as described above. Twelve DEGs involved in the interaction between the culture filtrate of *C. subaffine* LB-1 and *B. maydis* were selected for qRT-PCR analysis using a 7,500 Fast Real-Time PCR system (Thermo Fisher, Germany). Primers for the selected DEGs and the reference gene glyceraldehyde-3-phosphate dehydrogenase (*GAPDH*) (Sun et al., 2020) are listed in Supplementary Table 1. Three biological replicates were performed for each treatment sample (Bip_LB) and control sample (Bip_CK). Relative expression levels were calculated using the 2^−ΔΔCT^ method (Arocho et al., 2006).

### 2.6 Statistical analysis

Graphs for qRT-PCR validation were generated and statistically analyzed using GraphPad Prism (v. 8.0.2). Statistical analyses of the remaining experimental data were performed using Microsoft Excel 2016 and SPSS software. Differences between treatments were evaluated using Student’s *t*-test with *p* ≤ 0.05 regarded as statistically significant.

## 3 Results

### 3.1 Inhibitory effect of the culture filtrate of *C. subaffine* LB-1 on *B. maydis*

**Figure 1:**
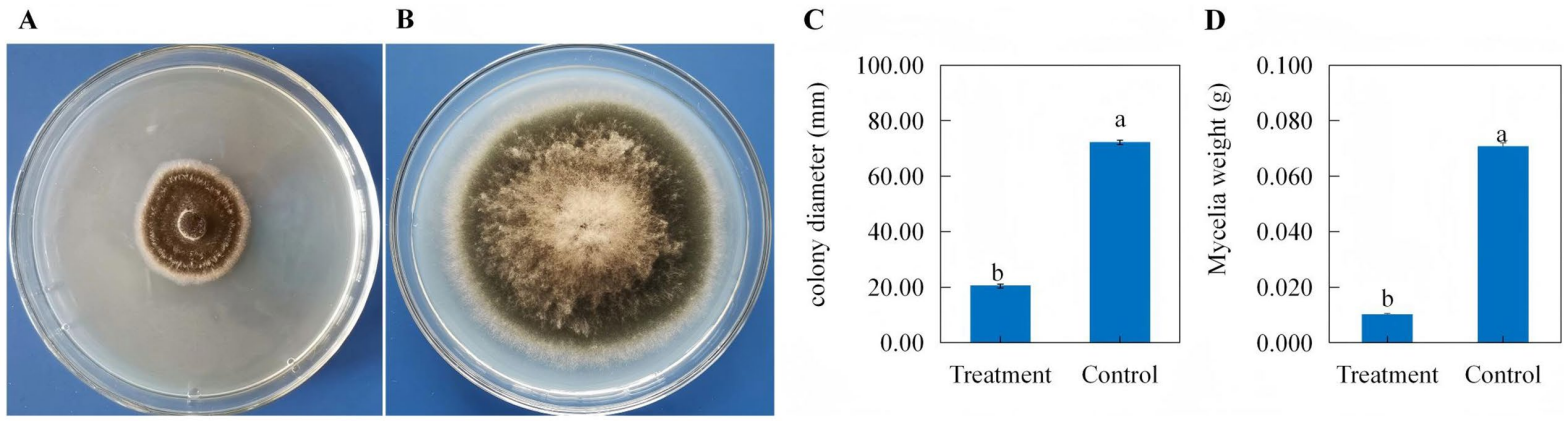
Growth of *Bipolaris maydis* cultured on PDA plates with or without the culture filtrate of *C. subaffine* LB-1 after 6 days of incubation. **(A)** *B. maydis* cultured on PDA plate amended with the culture filtrate. **(B)** *B. maydis* cultured on control PDA plates. **(C)** Colony diameter of *B. maydis* on PDA plates amended with the culture filtrate (treatment) and on normal PDA plates (control). **(D)** Mycelia weight of *B. maydis* on PDA plates amended with the culture filtrate (treatment) and normal PDA plates (control). Alt text: Graphs and data showed the effect of the culture filtrate of *C. subaffine* LB-1 on the colony extension and mycelia weight of the pathogenic fungus *B. maydis* using poison plate assay.

The poison plate assay demonstrated that the colony extension of *B. maydis* on treated PDA plates containing 1/3 (v/v) culture filtrate of *C. subaffine* LB-1 (Figure 1A) was significantly inhibited compared with that on control PDA plates (Figure 1B), with an inhibitory rate of 71.99% (Figure 1C). Consistently, the mycelial weight of *B. maydis* grown on the treated PDA plates was significantly lower than that on the control plates (Figure 1D). These results indicate that the culture filtrate of *C. subaffine* LB-1 exhibit a significant inhibitory effect on *B. maydis*.

The potted maize experiment further showed that compared with the disease index of southern corn leaf blight in control plants sprayed with distilled water, the highest control efficacy of 71.34% was achieved when maize plants were sprayed with the three-fold diluted culture filtrate of *C. subaffine* LB-1 both before and after inoculation. The disease index of southern corn leaf blight in maize plants sprayed with culture filtrate after inoculation was also significantly lower than that in control mazie plants (*p* ≤ 0.05), with a control efficacy of 63.94%. However, treatment applied only before inoculation resulted in a 30.72% control efficacy. No significant difference in the disease index was detected between maize plants treated with PDB and those treated with distilled water (Table 1). These results indicate that foliar application of the culture filtrate of *C. subaffine* LB-1 provide effective control of southern corn leaf blight caused by *B. maydis.*

**Table 1:** Control efficacy of the culture filtrate of *C. subaffine* LB-1 in potted maize plants.

Treatment	Disease index	Control efficacy (%)
Foliar spray prior to inoculation	11.60	30.72
Foliar spray post inoculation	6.04	63.94
Foliar spray pre- and post-inoculation	4.80	71.34
Foliar spray with PDB	15.79	5.68
Foliar spray with distilled water (control)	16.75	/

### 3.2 Mycolytic effect of the culture filtrate of *C. subaffine* LB-1 on *B. maydis*

**Figure 2:**
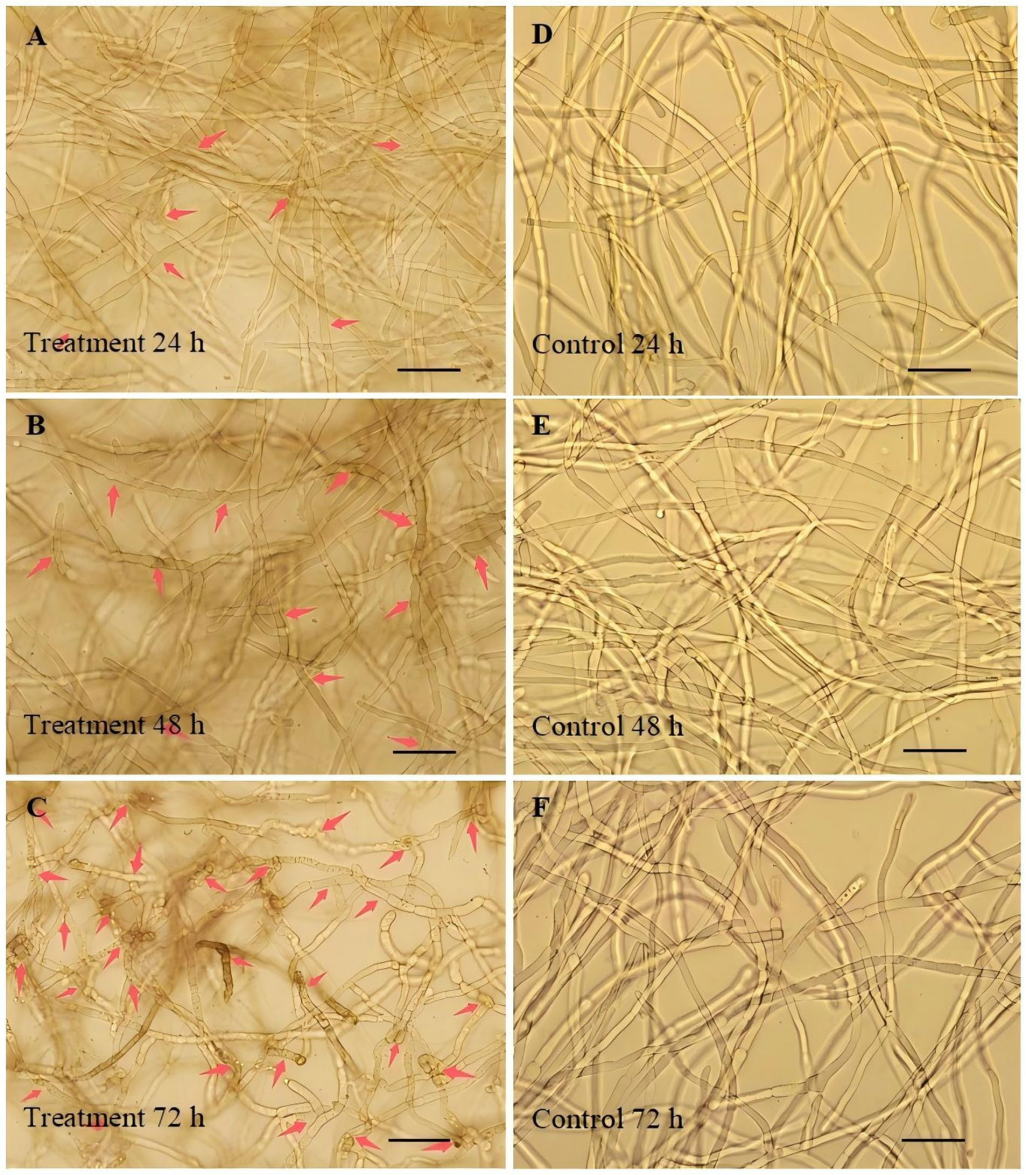
Morphological changes in *Bipolaris maydis* hyphae in response to the culture filtrate of *C. subaffine* LB-1 (treatment) and PDB (control). **(A–C)** Hyphae of *B. maydis* cultured in the culture filtrate of *C. subaffine* LB-1 (treatment) for 24, 48, and 72 h showing swollen and distorted morphology. Red arrows indicate aberrant hyphae. **(D–F)** Hyphae of *B. maydis* cultured in PDB (control) for 24, 48, and 72 h showing normal smooth and tubular morphology. Scale bar = 20 μm. Alt text: Graphs represents that *B. maydis* hyphae treated with the culture filtrate of *C. subaffine* LB-1 exhibited swelling and distortion that increased over time, whereas the control hyphae remained smooth and tubular.

Microscopic observations found that the hyphae of *B. maydis* became swollen after 24 h of incubation in the three-fold diluted culture filtrate of *C. subaffine* LB-1, and the swelling became more pronounced with prolonged incubation time (Figures 2A–C). In contrast, *B. maydis* cultured in PDB maintained normal, smooth, and tubular morphology throughout the observation period (Figures 2D–F). These results indicate that the culture filtrate of *C. subaffine* LB-1 induced abnormal hyphal morphology in *B. maydis*.

**Figure 3:**
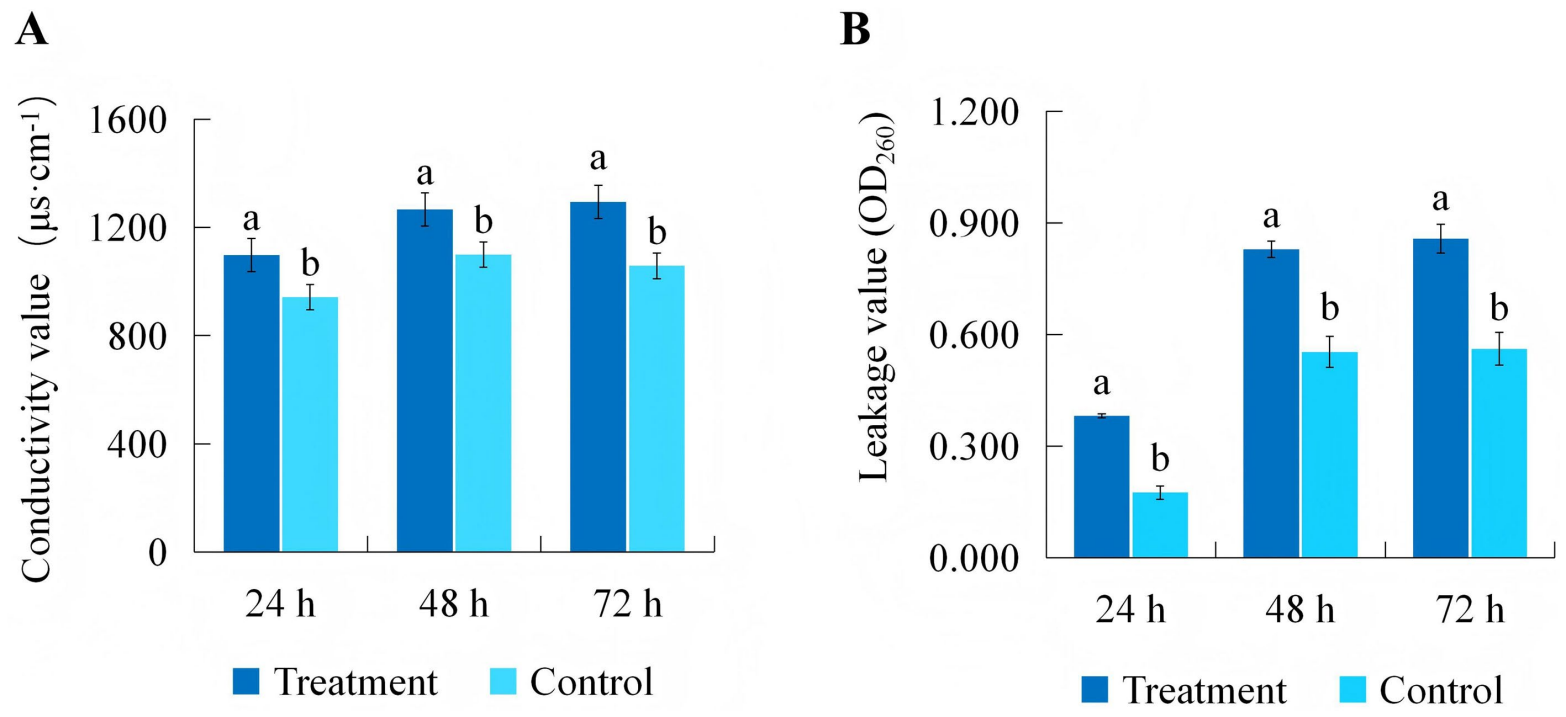
Electrical conductivity **(A)** and leakage value **(B)** of culture supernatants from *Bipolaris maydis* grown in the culture filtrate of *C. subaffine* LB-1 (treatment) and in PDB (control) for 24, 48, and 72 h individually. Vertical bars on the columns indicate the standard deviations of the mean (*n* = 3). Different lowercase letters indicate significant differences between treatment and control at the same time point (*p* ≤ 0.05). Alt text: Conductivity and leakage values in the culture supernatants of *B. maydis* were higher in the culture filtrate treatment than in the control at all-time points (24, 48, and 72 h), indicating increased membrane permeability.

Cell membrane permeability analysis further indicated that the conductivity and leakage value of *B. maydis* cultures grown in the three-fold diluted culture filtrate of *C. subaffine* LB-1 were significantly higher than those of the control cultures grown in PDB over the same period (Figure 3). These results indicate that the culture filtrate of *C. subaffine* LB-1 increases the cell membrane permeability of *B. maydis*, leading to the leakage of cellular contents.

### 3.3 Transcriptome analysis of Bip_LB vs. Bip_CK

#### 3.3.1 Data processing and quality assessment

RNA-seq analysis of six *B. maydis* samples, including three culture filtrate-treated group (Bip_LB) and three control group (Bip_CK), generated 41.39 G of raw reads. After removing reads containing adapters, poly N sequences, and low-quality bases, 39.87 G of clean reads were obtained. The Q20 value, defined as the percentage of bases with a mismatch probability of 1%, and the Q30 value, defined as the percentage of bases with a mismatch probability of 0.1%, remained high, exceeding 96.70 and 91.47%, respectively. The GC content of the clean reads ranged from 54.24 to 54.81%. Moreover, the total alignment rate of the clean reads exceeded 94.73%, the unique alignment rate exceeded 94.55%, and the proportion of properly aligned reads exceeded 90.99% (Supplementary Table 2). These results indicate that the transcriptomic data were of sufficient quality for subsequent analyses.

#### 3.3.2 DEGs, GO function, and KEGG pathway analyses

RNA-seq analysis identified a total of 4,124 DEGs in the Bip_LB and Bip_CK comparison (|log_2_FoldChange| ≥ 1 and padj ≤ 0.05), including 1,924 upregulated genes and 2,200 downregulated genes (Supplementary Figure 1).

**Figure 4:**
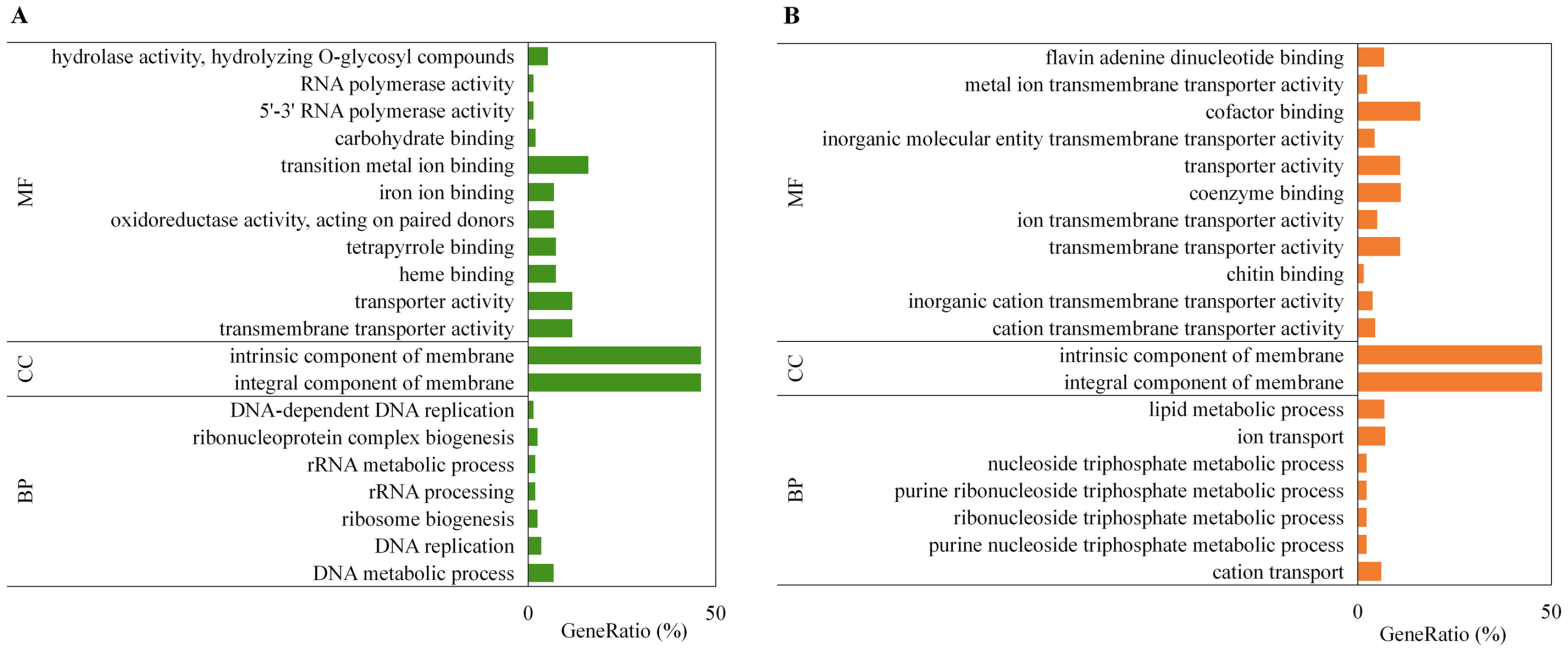
GO enrichment analysis of DEGs in Bip_LB vs. Bip_CK. **(A)** GO terms significantly enriched (*padj* ≤ 0.05) among downregulated DEGs. **(B)** GO terms significantly enriched (*padj* ≤ 0.05) among upregulated DEGs. The *y*-axis represents the GO terms, and the *x*-axis represents the ratio of DEGs annotated to each GO term. MF, Molecular function; CC, cellular component; BP, biological process. Alt text: Graphs showing GO enrichment differences in functional categories between upregulated and downregulated genes in *B. maydis* treated with the culture filtrate compared with the control.

GO enrichment analysis was performed to characterize the functions of the DEGs. Among the top 20 significantly enriched GO terms for the downregulated DEGs (padj ≤ 0.05), 11 terms belonged to the molecular function (MF) category, including hydrolase activity, RNA polymerase activity, binding, oxidoreductase activity, and transporter activity. Moreover, two terms belonged to the cellular component (CC) category and were associated with membrane components, and seven terms belonged to the biological process (BP) category, including DNA metabolism, rRNA metabolism, ribonucleoprotein complex biogenesis, and ribosome biogenesis (Figure 4A). Among the top 20 significantly enriched GO terms for the upregulated DEGs (padj ≤ 0.05), 11 terms belonged to the MF category, mainly transmembrane transporter activity and binding; two terms belonged to the CC category and were also associated with membrane components; and seven terms belonged to the BP category, including lipid metabolic process, ion transport, and ribonucleoside triphosphate metabolic process (Figure 4B). Notably, the GO terms intrinsic component of membrane (GO:0031224), integral component of membrane (GO:0016021), and transmembrane transporter activity (GO:0022857) were significantly enriched (padj ≤ 0.05) in both upregulated and downregulated DEG sets, indicating bidirectional functions and transporter activity of membrane component-related transporters.

**Figure 5:**
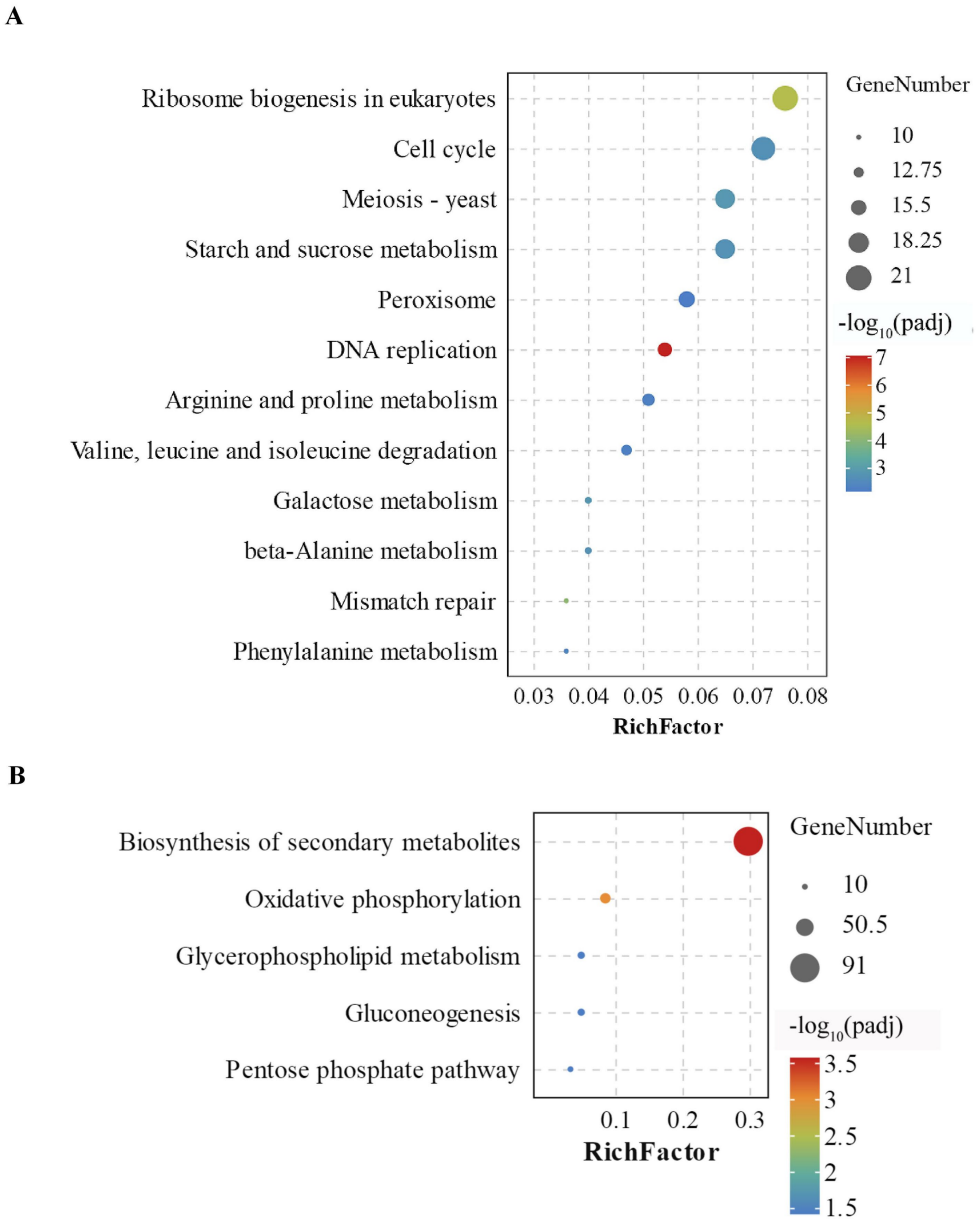
KEGG pathway enrichment analysis of DEGs in Bip_LB vs. Bip_CK. **(A)** KEGG pathways significantly enriched (*padj* ≤ 0.05) among downregulated DEGs. **(B)** KEGG pathways significantly enriched (*padj* ≤ 0.05) among upregulated DEGs. RichFactor represents the ratio of DEGs enriched in a given KEGG pathway. The dot size represents the number of DEGs enriched in each pathway. Colors from red to purple indicate enrichment significance. *Padj* ≤ 0.05 was used as the threshold of significance. Alt text: Graphs showing the metabolic pathways and enrichment levels of the upregulated and downregulated genes enriched in *B. maydis* treated with the culture filtrate of *C. subaffine* LB-1 vs. control treated with PDB.

KEGG pathway enrichment analysis was performed to identify the major metabolic pathways involving DEGs. The downregulated DEGs were significantly enriched (padj ≤ 0.05) in 12 pathways, including ribosome biogenesis, cell cycle, meiosis, DNA replication, mismatch repair, carbohydrate metabolism, and amino acid metabolism (Figure 5A). In contrast, the upregulated DEGs were significantly enriched (padj ≤ 0.05) in five pathways, including biosynthesis of secondary metabolites, oxidative phosphorylation, glycerophospholipid metabolism, gluconeogenesis, and the pentose phosphate pathway (Figure 5B).

#### 3.3.3 DEGs involved in the interaction of the culture filtrate of *C. subaffine* LB-1 with *B. maydis*

Based on the results of GO functional annotation and KEGG pathway enrichment analyses, DEGs associated with transmembrane transport, carbohydrate metabolism, amino acid metabolism, cell proliferation, and cellular detoxification were further analyzed. The results demonstrated that 60 DEGs involved in nutrient transmembrane transport, including genes annotated as sugar transporter, ammonium transporter, sterol carrier protein, calcium-channel protein, sodium transport ATPase, and fatty acid transport protein, together with DEGs involved in carbohydrate degradation (e.g., glycoside hydrolase and dehydrogenase), amino acid degradation (e.g., decarboxylase and aminotransferase), and cell proliferation (e.g., DNA polymerase, RNA polymerase, and ribonuclease), were significantly downregulated (Table 2). In contrast, 40 DEGs associated with lipid degradation (e.g., phospholipase), the pentose phosphate pathway (e.g., 6-phosphogluconate dehydrogenase), secondary metabolism related to detoxification (e.g., cytochrome P450 monooxygenase and FAD-dependent monooxygenase), and drug efflux (e.g., ATP-binding cassette (ABC) multidrug transporter, major facilitator superfamily (MFS) multidrug transporter, and efflux pump), were significantly upregulated (Table 3).

**Table 2:** Downregulated DEGs associated with the antifungal activity of the culture filtrate of *C. subaffine* LB-1.

Gene	log2FoldChange	*Padj*	Gene annotation
*COCC4DRAFT_63270*	−1.660000038	8.24 × 10^−3^	High-affinity fructose transporter
*COCC4DRAFT_180711*	−1.303368975	2.18 × 10^−8^	High-affinity fructose transporter
*COCC4DRAFT_190734*	−2.295589473	1.21 × 10^−37^	Glucose/galactose transporter
*COCC4DRAFT_208374*	−3.516837802	4.04 × 10^−24^	Ammonium transporter
*COCC4DRAFT_63030*	−2.602737725	3.15 × 10^−22^	Sugar transporter
*COCC4DRAFT_188092*	−1.230820621	2.10 × 10^−7^	Calcium-channel protein
*COCC4DRAFT_143946*	−1.885285874	5.97 × 10^−23^	Sterol carrier protein
*COCC4DRAFT_37803*	−2.684340351	7.95 × 10^−43^	Calcium-transporting ATPase
*COCC4DRAFT_19500*	−2.044676719	2.92 × 10^−7^	Sodium transport ATPase
*COCC4DRAFT_161772*	−2.676994874	5.43 × 10^−14^	Very long-chain fatty acid transport protein
*COCC4DRAFT_189412*	−3.374033938	2.04 × 10^−59^	Glycoside hydrolase family
*COCC4DRAFT_179858*	−3.403012559	1.18 × 10^−44^	Glycoside hydrolase family
*COCC4DRAFT_181602*	−3.637633024	8.82 × 10^−24^	Glycoside hydrolase family
*COCC4DRAFT_133665*	−2.071331975	6.35 × 10^−14^	Glycoside hydrolase family
*COCC4DRAFT_202015*	−1.384949783	1.82 × 10^−12^	Glycoside hydrolase family
*COCC4DRAFT_61261*	−1.265201679	4.03 × 10^−11^	Glycoside hydrolase family
*COCC4DRAFT_165196*	−1.506512248	2.03 × 10^−10^	Glycoside hydrolase family
*COCC4DRAFT_198270*	−1.561617897	1.64 × 10^−9^	Glycoside hydrolase family
*COCC4DRAFT_29785*	−1.083755183	4.59 × 10^−8^	Glycoside hydrolase family
*COCC4DRAFT_173115*	−1.144336208	2.22 × 10^−7^	Glycoside hydrolase family
*COCC4DRAFT_28016*	−1.269995578	2.31 × 10^−6^	Glycoside hydrolase family
*COCC4DRAFT_69079*	−1.880434249	8.72 × 10^−6^	Glycoside hydrolase family
*COCC4DRAFT_55244*	−1.136178437	4.12 × 10^−5^	Glycoside hydrolase family
*COCC4DRAFT_67579*	−1.659655189	1.63 × 10^−4^	Glycoside hydrolase family
*COCC4DRAFT_43111*	−1.134979949	5.95 × 10^−4^	Glycoside hydrolase family
*COCC4DRAFT_208489*	−1.033811814	1.28 × 10^−3^	Glycoside hydrolase family
*COCC4DRAFT_18610*	−5.543685859	5.55 × 10^−46^	Dehydrogenase
*COCC4DRAFT_192351*	−4.241299856	6.55 × 10^−30^	Dehydrogenase
*COCC4DRAFT_137389*	−4.183460367	1.87 × 10^−17^	Dehydrogenase
*COCC4DRAFT_191604*	−1.579287977	1.07 × 10^−14^	Dehydrogenase
*COCC4DRAFT_54262*	−1.020881373	5.29 × 10^−3^	Dehydrogenase
*COCC4DRAFT_49774*	−1.382106312	4.06 × 10^−2^	Dehydrogenase
*COCC4DRAFT_66390*	−4.411829908	7.14 × 10^−19^	Decarboxylase
*COCC4DRAFT_61291*	−1.108384664	1.17 × 10^−15^	Decarboxylase
*COCC4DRAFT_180695*	−1.470947689	3.81 × 10^−9^	Decarboxylase
*COCC4DRAFT_185126*	−2.001939776	8.52 × 10^−5^	Decarboxylase
*COCC4DRAFT_144410*	−1.00729957	3.37 × 10^−4^	Decarboxylase
*COCC4DRAFT_49107*	−1.639611467	9.43 × 10^−3^	Decarboxylase
*COCC4DRAFT_59394*	−2.081899989	5.85 × 10^−3^	Aminotransferase
*COCC4DRAFT_140884*	−1.738151789	6.65 × 10^−3^	Aminotransferase
*COCC4DRAFT_191575*	−1.304744278	1.94 × 10^−8^	Aminotransferase
*COCC4DRAFT_129504*	−4.803598991	8.58 × 10^−20^	Aminotransferase
*COCC4DRAFT_130779*	−1.535568538	2.96 × 10^−15^	RNA-binding protein
*COCC4DRAFT_180267*	−1.738589232	3.85 × 10^−17^	DNA polymerase epsilon subunit B
*COCC4DRAFT_199477*	−1.708210366	1.73 × 10^−16^	DNA polymerase
*COCC4DRAFT_73563*	−1.338612387	3.67 × 10^−13^	DNA polymerase
*COCC4DRAFT_186425*	−1.194648167	2.27 × 10^−11^	DNA polymerase
*COCC4DRAFT_166165*	−1.214234648	1.07 × 10^−10^	DNA polymerase
*COCC4DRAFT_205987*	−1.145806757	1.20 × 10^−9^	Polymerase II transcription subunit
*COCC4DRAFT_143118*	−1.240497102	5.70 × 10^−9^	RNA polymerase III subunit
*COCC4DRAFT_169011*	−1.099206764	2.48 × 10^−7^	DNA polymerase
*COCC4DRAFT_181193*	−1.237265955	1.41 × 10^−6^	RNA polymerase III subunit
*COCC4DRAFT_71038*	−1.022200402	4.27 × 10^−6^	RNA polymerase III subunit
*COCC4DRAFT_58575*	−1.158894263	1.19 × 10^−5^	RNA polymerases I and III subunit
*COCC4DRAFT_57225*	−1.050434523	1.23 × 10^−4^	DNA polymerase
*COCC4DRAFT_136830*	−1.216452271	1.28 × 10^−2^	DNA polymerase
*COCC4DRAFT_122705*	−1.304663657	4.57 × 10^−4^	Ribonuclease
*COCC4DRAFT_192354*	−2.87641425	3.47 × 10^−29^	Ribonuclease
*COCC4DRAFT_148955*	−5.514553646	9.65 × 10^−27^	Ribonuclease
*COCC4DRAFT_148247*	−2.222165411	1.69 × 10^−15^	Ribonuclease

**Table 3:** Upregulated DEGs associated with the stress response of *B. maydis.*

Gene	log2FoldChange	*Padj*	Gene annotation
*COCC4DRAFT_83490*	1.818834351	2.78 × 10^−34^	Phospholipase
*COCC4DRAFT_20549*	1.46971147	1.12 × 10^−22^	Phospholipase
*COCC4DRAFT_19893*	2.676379066	1.23 × 10^−19^	Phospholipase
*COCC4DRAFT_59580*	2.067608085	6.02 × 10^−8^	Phospholipase
*COCC4DRAFT_181456*	2.319224435	1.70 × 10^−6^	Phospholipase
*COCC4DRAFT_170867*	1.077897434	1.05 × 10^−3^	Phospholipase
*COCC4DRAFT_158033*	1.101459366	3.79 × 10^−3^	Phospholipase
*COCC4DRAFT_122711*	1.676998155	7.58 × 10^−13^	6-phosphogluconate dehydrogenase
*COCC4DRAFT_31870*	2.194393604	1.06 × 10^−26^	Cytochrome P450 monooxygenase
*COCC4DRAFT_128869*	2.22664361	4.38 × 10^−23^	Cytochrome P450 monooxygenase
*COCC4DRAFT_173722*	3.416718636	1.51 × 10^−18^	Cytochrome P450 monooxygenase
*COCC4DRAFT_171292*	4.195439713	8.72 × 10^−15^	Cytochrome P450 monooxygenase
*COCC4DRAFT_127527*	3.619912012	1.49 × 10^−12^	Cytochrome P450 monooxygenase
*COCC4DRAFT_46259*	2.004082217	4.60 × 10^−11^	Cytochrome P450 monooxygenase
*COCC4DRAFT_169587*	1.526131793	1.67 × 10^−5^	Cytochrome P450 monooxygenase
*COCC4DRAFT_173190*	2.241041623	2.64 × 10^−4^	Cytochrome P450 monooxygenase
*COCC4DRAFT_19072*	1.865207798	2.50 × 10^−3^	Cytochrome P450 monooxygenase
*COCC4DRAFT_81703*	1.91457297	3.34 × 10^−13^	FAD-dependent monooxygenase
*COCC4DRAFT_75311*	2.647145694	9.37 × 10^−11^	FAD-dependent monooxygenase
*COCC4DRAFT_182117*	1.120104797	7.23 × 10^−8^	FAD-dependent monooxygenase
*COCC4DRAFT_124440*	1.832565443	7.12 × 10^−5^	FAD-dependent monooxygenase
*COCC4DRAFT_79153*	1.461742144	1.09 × 10^−3^	FAD-dependent monooxygenase
*COCC4DRAFT_77695*	1.448024451	9.70 × 10^−3^	FAD-dependent oxidoreductase
*COCC4DRAFT_32528*	6.30459276	6.53 × 10^−77^	MFS multidrug transporter
*COCC4DRAFT_197028*	2.509297654	2.19 × 10^−22^	MFS-type transporter
*COCC4DRAFT_80407*	2.268685333	7.34 × 10^−20^	MFS-type transporter
*COCC4DRAFT_161419*	3.773204515	8.81 × 10^−16^	MFS multidrug transporter
*COCC4DRAFT_196446*	1.627105295	1.74 × 10^−10^	MFS multidrug transporter
*COCC4DRAFT_73657*	3.634378534	1.48 × 10^−7^	MFS-type transporter
*COCC4DRAFT_137273*	1.330804261	1.41 × 10^−5^	MFS multidrug transporter
*COCC4DRAFT_51499*	4.326547799	1.98 × 10^−66^	Major Facilitator Superfamily
*COCC4DRAFT_67007*	3.810260001	2.28 × 10^−17^	Major Facilitator Superfamily
*COCC4DRAFT_60558*	1.596356104	2.46 × 10^−12^	Major Facilitator Superfamily
*COCC4DRAFT_61165*	2.750722628	5.30 × 10^−7^	Major Facilitator Superfamily
*COCC4DRAFT_209248*	3.119075536	7.37 × 10^−37^	ABC transporter
*COCC4DRAFT_170826*	1.34226257	3.31 × 10^−20^	ABC multidrug transporter
*COCC4DRAFT_57728*	2.434818878	4.33 × 10^−11^	ABC multidrug transporter
*COCC4DRAFT_191368*	2.105520614	1.93 × 10^−7^	ABC multidrug transporter
*COCC4DRAFT_45517*	2.534432788	1.67 × 10^−15^	Efflux pump
*COCC4DRAFT_191496*	1.585006427	7.69 × 10^−15^	Efflux pump

#### 3.3.4 Validation of DEGs by qRT-PCR

**Figure 6:**
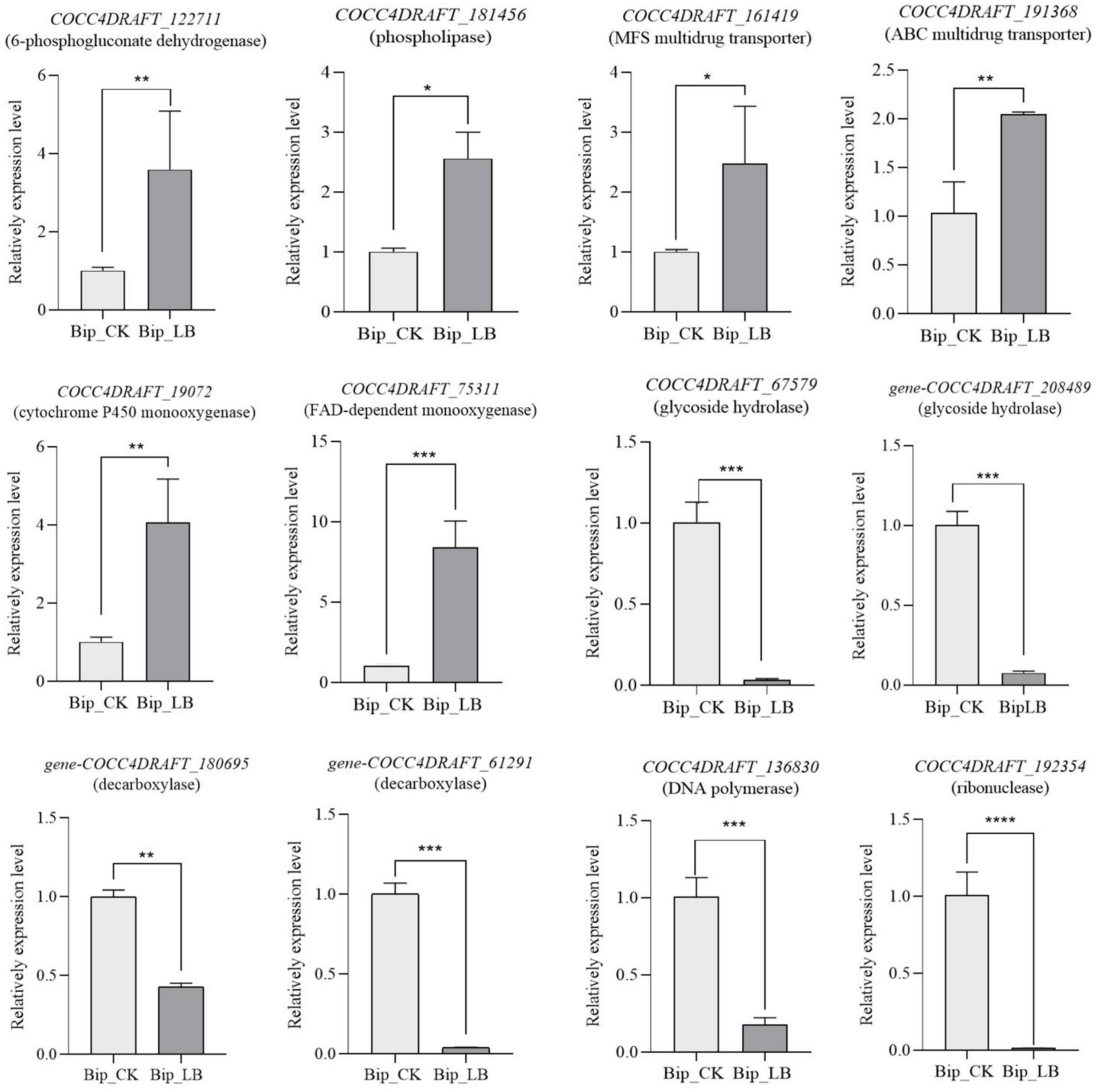
qRT-PCR validation of selected DEGs in Bip_LB vs. Bip_CK. Error bars represent the standard deviation (*n* = 3). Asterisks indicate significant difference between Bip_CK (control) and Bip_LB (treatment) determined by Student’s *t*-test (*p ≤ 0.05; **p ≤ 0.01; ***p ≤ 0.001; ****p ≤ 0.0001). Alt text: Bar graphs showing expression of selected DEGs between Bip_LB and Bip_CK by qRT-PCR, with significant differences observed between treatment and control groups.

qRT-PCR assay showed that the tested DEGs exhibited expression patterns consistent with those of the RNA-seq results. Specifically, genes encoding 6-phosphogluconate dehydrogenase (*COCC4DRAFT_122711*), phospholipase (*COCC4DRAFT_181456*), MFS multidrug transporter (*COCC4DRAFT_161419*), ABC multidrug transporter (*COCC4DRAFT_191368*), cytochrome P450 monooxygenase (*COCC4DRAFT_19072*), and FAD-dependent monooxygenase (*COCC4DRAFT_75311*) were upregulated. Additionally, the genes encoding glycoside hydrolase (*COCC4DRAFT_67579* and *COCC4DRAFT_208489*), decarboxylase (*COCC4DRAFT_180695* and *COCC4DRAFT_61291*), DNA polymerase (*COCC4DRAFT_136830*), and ribonuclease (*COCC4DRAFT_192354*) were downregulated (Figure 6). These quantitative results were consistent with the RNA-seq analysis.

## 4 Discussion

*Chaetomium* spp. are fungi capable of producing a wide range bioactive compounds (Gao et al., 2023; Dwibedi et al., 2023). Among biocontrol *Chaetomium* species, the antifungal activity of culture filtrate of *C. globosum* has been widely reported (Zhao et al., 2017; Linkies et al., 2021; Spinelli et al., 2025). In contrast, the antifungal activity of the culture filtrate of other *Chaetomium* spp., as well as their mechanisms of action, have received less attention. This study confirmed that the culture filtrate of *C. subaffine* LB-1 exhibited antifungal and mycolytic effects against the phytopathogenic fungus *B. maydis*. The culture filtrate impaired cell membrane permeability, resulting in leakage of cellular contents and disruption of nutrient uptake and metabolism in *B. maydis*. In response, metabolic processes related to nutrient and energy compensation and detoxification were activated in the pathogen.

Mycolysis is a mode of action by which biocontrol strains act against phytopathogenic fungi and is typically characterized by inhibited growth and swollen or even lysed hyphae in the target fungus (Aktuganov et al., 2008). Accordingly, many studies have attributed this mycolytic effect to impaired cell membrane permeability in the pathogen, which can be evaluated by measuring the conductivity and leakage values of the pathogen culture supernatant (Xu et al., 2019; Li et al., 2022). In this study, swollen hyphae, along with increased conductivity and leakage values in the culture supernatant of *B. maydis* shake-cultured in the culture filtrate of *C. subaffine* LB-1, were detected. These results indicate that the culture filtrate of *C. subaffine* LB-1 exerts a mycolytic effect on *B. maydis*.

The antifungal activities of culture filtrate from biocontrol strains may result from the production of antibiotic substances and cell wall-degrading enzymes (Al-Askar et al., 2015; Li et al., 2022; Yi et al., 2022). Currently, antifungal metabolites such as chaetoglobosin (Zhang et al., 2013), chaetoviridin (Yan et al., 2018), chaetogline (Yan et al., 2019), and benzothiazole (Sangeetha et al., 2024) have been identified in *C. globosum*. In the present study, strong antifungal activity of *B. maydis* caused by the culture filtrate of *C. subaffine* LB-1 were detected, this activity may be the action of hydrolase, antifungal substance, or a combination of both components present in the culture filtrate. Therefore, further studies are required to identify the specific antifungal components contained in the culture filtrate of *C. subaffine* LB-1.

Carbohydrates and amino acids are essential nutrients for cellular metabolism and growth, and their uptake is primarily mediated by specific membrane proteins that function as nutrient transporters (Edinger, 2007). Under nutrient-limited conditions, however, the degradation of lipid reserves can contribute to nutrient supply in fungi (Ghannoum, 2000; Thines et al., 2000). Transcriptome analysis in the present study showed that genes involved in nutrient transport, including fructose transporter, glucose transporter, and ammonium transporter, as well as genes associated with carbohydrate and amino acid degradation, (e.g., glycoside hydrolase, decarboxylase, and aminotransferase), were significantly downregulated in *B. maydis* treated with the culture filtrate of *C. subaffine* LB-1compared with control *B. maydis* (Table 2); In contrast, DEGs annotated as phospholipase were upregulated (Table 3). In addition, the GO term hydrolase activity (hydrolyzing O-glycosyl compounds) (Figure 4A) and KEGG pathways sucrose and starch metabolism, galactose metabolism, and amino acid metabolism were significantly downenriched (Figure 5A). Conversely, the GO term lipid metabolic process (Figure 4B) and the KEGG pathway glycerophospholipid metabolism (Figure 5B) were significantly upenriched. These results suggest that the culture fitrate of *C. subaffine* LB-1may affect cell membrane permeability of *B. maydis*, which could be associated with reduced nutrient uptake and decreased carbohydrate and amino acid metabolism. The upregulated genes and upenriched pathways related with lipid metabolism may indicate a compensatory response of *B. maydis* under these conditions.

Glycolysis is the principal pathway for carbohydrate degradation and energy production in cells. However, the pentose phosphate pathway, which branches from glycolysis at glucose-6-phosphate contributes to cellular metabolism by generating reducing power in the form of NADPH (Jeppsson et al., 2002; Wamelink et al., 2008). In this study, RNA-seq analysis showed that the gene encoding 6-phosphogluconate dehydrogenase was significantly upregulated (Table 3), and the pentose phosphate pathway was significantly upenriched in *B. maydis* treated with the culture filtrate of *C. subaffine* LB-1(Figure 5B). These results suggest that the pentose phosphate pathway may be involved in the metabolic response of *B. maydis* upon exposure to the culture filtrate of *C. subaffine* LB-1.

In fungi, FAD-dependent monooxygenases and cytochrome P450 monooxygenases drive redox reactions associated with the biosynthesis of secondary metabolites, including those related to detoxification (Heine et al., 2018). In addition, ABC and MFS transporters mediate the active cellular efflux of toxic compounds (Spios and Kuchler, 2006; Utami et al., 2020). In this study, RNA-seq analysis revealed that 33 DEGs annotated as FAD-containing monooxygenases, cytochrome P450 monooxygenases, ABC multidrug transporters, and MFS multidrug transporters were significantly upregulated in *B. maydis* treated with the culture filtrate of *C. subaffine* LB-1 compared with the control *B. maydis* (Table 3). In addition, GO terms related to transmembrane transport activities (Figure 4B) and the KEGG pathway of biosynthesis of secondary metabolites were significantly upenriched (Figure 5B). These findings suggest that detoxification metabolism and drug efflux were activated in *B. maydis* under the stress of the culture filtrate of *C. subaffine* LB-1.

Taken together, this study confirmed the antifungal activity and mycolytic effect of the culture filtrate of *C. subaffine* LB-1 on plant pathogenic fungus *B. maydis*, elucidated the antifungal mechanism of the culture filtrate and the stress response activated in the pathogen. However, gene expression level is not necessarily indicative of its functional activity. Thus, the molecular mechanism of the culture filtrate of *C. subaffine* LB-1 against plant pathogenic fungi need to be further identified using gene knockdown, proteomic or metabolomic, etc.

## Data Availability

The datasets presented in this study can be found in online repositories. The names of the repository/repositories and accession number(s) can be found in the article/Supplementary material.
