# Supplementary material for: Antifungal activity of the culture filtrate of Chaetomium subaffine LB-1 against Bipolaris maydis and its underlying interaction mechanism
Source: Front Microbiol. 2026 May 20;17:1848272. doi: 10.3389/fmicb.2026.1848272 (PMC13230150; doi:10.3389/fmicb.2026.1848272)
Supplement: Supplementary file 1 [file Table_1.docx]

Supplementary Material

# Supplementary Table 1 Primers for qRT-PCR validation of DEGs.

| **Gene** | **Forward primer (5′-3′)** | **Reverse primer (5′-3′)** |
| --- | --- | --- |
| *COCC4DRAFT_122711* | TGTTGGTGTCATTGGCAAGG | TCTCCAATCTCCTCATCGCC |
| *COCC4DRAFT_181456* | TGCTGCTCTCACCTGTAACA | ATGTCATGTCCGCAGCATTC |
| *COCC4DRAFT_161419* | TGTGTTCATTATGCGCGGAG | CCATTCCAAAGGATCCGCAG |
| *COCC4DRAFT_191368* | GATTCTTGTTCTCGGACGCC | TACTCATCTCGTCCGCATCC |
| *COCC4DRAFT_19072* | GCTGGGCTGAGATCGAAAAG | TTGGGGAAGATCTTGGGGTC |
| *COCC4DRAFT_75311* | CGTGGGCATGATGTCTTTGT | CCGTGTTTGATTTGGCTTGC |
| *COCC4DRAFT_67579* | GGTCCTTGTGCTGTCTACCT | TCTCACAGAAAGAAGGCCGT |
| *COCC4DRAFT_208489* | CGTACAATGGCTTCAGTCCG | TCCAGAAACCCAGCACAGAT |
| *COCC4DRAFT_180695* | CCCTGCACTATCCGGATCAT | CGTCGACCAAGAATAGCACG |
| *COCC4DRAFT_61291* | GGTTTGAGGAGCACAAGGGT | TCTGCACCGATCACAGCTTC |
| *COCC4DRAFT_136830* | CCCCTCAACGCGATACCTAC | AATGTGCCATCAGCCTCCTC |
| *COCC4DRAFT_192354* | TGGCAGAAGCACAGGATGAC | TTGCGGCTTGGAAATTGACG |
| *GAPDH* | CAACGGCTTCGGTCGCATTG | GCCAAGCAGTTGGTTGTGC |
