# Supplementary material for: Antifungal activity of the culture filtrate of Chaetomium subaffine LB-1 against Bipolaris maydis and its underlying interaction mechanism
Source: Front Microbiol. 2026 May 20;17:1848272. doi: 10.3389/fmicb.2026.1848272 (PMC13230150; doi:10.3389/fmicb.2026.1848272)
Supplement: Supplementary file 2 [file Table_2.docx]

Supplementary Material

# Supplementary Table 2 Sequencing and assembly quality of RNA-seq reads from six samples of *B. maydis*.

| **Sample** | **Raw reads** | **Clean reads** | **Q20 (%)** | **Q30 (%)** | **GC content (%)** | **Total mapped**  **(%)** | **Unique mapped**  **(%)** | **Proper mapped**  **(%)** |
| --- | --- | --- | --- | --- | --- | --- | --- | --- |
| Bip1_LB | 7.05G | 6.79G | 97.13 | 92.40 | 54.60 | 95.66 | 95.46 | 92.48 |
| Bip2_LB | 6.98G | 6.70G | 97.07 | 92.34 | 54.81 | 95.19 | 95.00 | 91.51 |
| Bip3_LB | 7.05G | 6.78G | 96.88 | 91.92 | 54.77 | 94.73 | 94.55 | 90.99 |
| Bip1_CK | 7.02G | 6.81G | 96.70 | 91.47 | 54.28 | 95.21 | 95.06 | 91.99 |
| Bip2_CK | 6.55G | 6.33G | 96.84 | 91.76 | 54.33 | 95.16 | 95.01 | 91.71 |
| Bip3_CK | 6.74G | 6.46G | 96.90 | 91.98 | 54.24 | 95.16 | 94.99 | 91.50 |

* Bip1_LB, Bip2_LB, and Bip3_LB represents three replicates of *B. maydis* treated with the culture filtrate of *C. subaffine* LB-1.

Bip1_CK, Bip2_CK, and Bip3_CK represents three replicates of the control *B. maydis.*
