# Supplementary material for: Antifungal activity of the culture filtrate of Chaetomium subaffine LB-1 against Bipolaris maydis and its underlying interaction mechanism
Source: Front Microbiol. 2026 May 20;17:1848272. doi: 10.3389/fmicb.2026.1848272 (PMC13230150; doi:10.3389/fmicb.2026.1848272)
Supplement: Supplementary file 3 [file Data_sheet_1.docx]

Supplementary Material


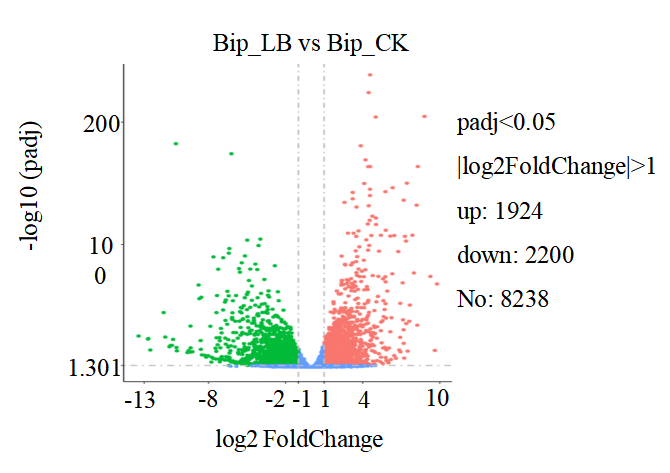


**Supplementary Figure 1.** Volcano plot of DEGs in Bip_LB vs. Bip_CK. The x-axis shows log2FoldChange (fold change in gene expression), and the y-axis shows −log10 (padj) (significance level). The red, green, and blue dots represent upregulated DEGs, downregulated DEGs, and the non-significant genes, respectively.
